# Childhood risk factors and clinical and service outcomes in adulthood in people with intellectual disabilities

**DOI:** 10.1192/bjo.2024.811

**Published:** 2024-12-04

**Authors:** B. Perera, S. Mufti, C. Norris, A. Baksh, V. Totsika, A. Hassiotis, P. Hurks, T. van Amelsvoort

**Affiliations:** Division of Psychiatry, University College London, UK; Hanringey LD Services, Barnet, Enfield and Haringey MH Trust, London, UK; Institute of Psychiatry, Psychology and Neuroscience, Kings College London, UK; School for Mental Health and Neuroscience, Maastricht University, Netherlands; Faculty of Psychology and Neuroscience, Maastricht University, Netherlands

**Keywords:** Intellectual disability, risk factors, challenging behaviour, attention-deficit hyperactivity disorder, mental health outcomes

## Abstract

**Background:**

Adults with intellectual disability experience increased rates of mental health disorders and adverse mental health outcomes.

**Aim:**

Explore childhood risk factors associated with adverse mental health outcomes during adulthood as defined by high cost of care, use of psychotropic medication without a severe mental illness and psychiatric hospital admissions.

**Method:**

Data on 137 adults with intellectual disability were collected through an intellectual disability community service in an inner London borough. Childhood modifiable and non-modifiable risk factors were extracted from records to map onto variables identified as potential risk factors. Logistic and linear regression models were employed to analyse their associations with adverse outcomes.

**Results:**

We showed that the co-occurrence of intellectual disability with autism spectrum disorder and/or attention-deficit hyperactivity disorder (ADHD) were associated with psychotropic medication use and high-cost care packages. However, when challenging behaviour during childhood was added, ADHD and autism spectrum disorder were no longer significant and challenging behaviour better explained medication prescribing and higher cost care. In addition, the severity of intellectual disability was associated with higher cost care packages. Ethnicity (Black and mixed) also predicted higher cost of care.

**Conclusions:**

Challenging behaviour during childhood emerged as a critical variable affecting outcomes in young adulthood and mediated the association between adult adverse mental health outcomes and co-occurring neurodevelopmental conditions, that is, ADHD and autism. These findings emphasise the need for effective early intervention strategies to address challenging behaviour during childhood. Such interventions for challenging behaviour will need to take into consideration autism and ADHD.

Intellectual disability is characterised by significant impairments in cognitive, social, functional and adaptive skills, including activities of daily living, with onset during the developmental period.^1,2^ Estimates suggest that approximately 1.5–2% of the population has intellectual disability.^3^ People with intellectual disability experience high rates of co-occurring mental health disorders and challenging behaviour compared to those without intellectual disability.^4^ Studies indicate that people with intellectual disability are three to four times more likely to have at least one other mental health disorder, such as mood disorders, anxiety disorders, schizophrenia and personality disorders, compared to individuals without intellectual disability.^5–8^ Challenging behaviours, that is, behaviours that place an individual or those in his environment at risk of harm or exclusion,^9^ are frequently observed among individuals with intellectual disability, prompting mental health services to provide assessments and interventions. It is estimated that about one in five people with intellectual disability known to mental health services present with challenging behaviour.^10^ Therefore, gaining a better understanding of the presentation and factors contributing to the increased risk of mental disorders and challenging behaviour in adults with intellectual disability is important to tackle this disparity in mental health among people with intellectual disability.^11,12^

## Risk factors

Studies focusing on the adult population without intellectual disability show multiple factors that increase the risk of mental illnesses. These include biological, psychological and social factors, such as genetics, gender, a history of previous mental illness, medical illnesses, substance misuse and childhood adversities.^13^ Studies in non-intellectual disability populations have demonstrated significant functional impairments, including mental illnesses among people with neurodevelopmental disorders such as attention-deficit hyperactivity disorder (ADHD).^14^ Individuals with intellectual disability face comparable risk factors associated with mental disorders and challenging behaviour.^15^ Communication impairments, lack of support systems, severity of intellectual disability and residing in institutional or congregate care settings are some factors specifically related to people with intellectual disability that contribute to increased mental health disorders.^16,17^

Studies have reported similar risk factors that increase the likelihood of challenging behaviour in intellectual disability. Factors such as the presence of a mental disorder, younger age, psychotropic medication use, pervasive developmental disorders, mood instability, agitation, irritability, increased contact with mental health professionals, underlying genetic disorders and sensory deficits are highly prevalent among people with intellectual disability experiencing challenging behaviour.^10^

## Clinical outcomes

Research indicates that individuals with intellectual disability often experience prolonged stays in psychiatric hospitals compared to those without intellectual disability.^18^ It is estimated that people with intellectual disability have 1.5 times longer hospital stays compared to people without.^19^ In addition, support required for individuals with intellectual disability with mental health or behavioural challenges demands increased resources, leading to significantly higher care costs in the community compared to those without intellectual disability.^20^ The presence of a mental disorder further increases the support needs and poses more demands on the care system.^21^ Studies have shown that people with intellectual disability are more likely to take psychotropic medications, including antipsychotic medications, even in the absence of severe mental illness.^22^ Hospital admissions, use of psychotropic medications in the absence of mental illness and higher cost of care could also be considered as important indicators of adverse mental health outcomes against the current service backdrop of limited availability of in-patient beds for people with intellectual disability, the financial costs of services supporting people with intellectual disability and the ongoing efforts to minimise the use of psychotropic medications.^23,24^

Studies in the general population have shown that up to three-quarters of mental disorders occur before age 25 years, emphasising the crucial need to address mental health concerns during this period.^25^ Similar findings have been reported in studies in people with intellectual disability suggesting that owing to reasons such as increased challenges when transitioning into adulthood, young adults (age 18–24 years) with intellectual disability often require access to intellectual disability mental health services.^26^ Therefore, focusing on the age group of 18–24 can be argued as important to be able to understand factors associated with clinical and service outcomes in young adults.

## Aim

Understanding risk factors for adverse mental health outcomes and their significance to clinical practice can prompt service improvements through preventive interventions that could drive changes at the service level, ultimately enhancing outcomes for individuals with intellectual disability. Considering the above, our study aimed to examine the association between childhood risk factors and adverse mental health outcomes in young adults with intellectual disability.

## Method

Data were drawn from electronic records of adults with intellectual disability aged 18–24 years registered with an intellectual disability service for adults in an inner London borough with a population of 280 000. There were approximately 700 adults with intellectual disability registered with the service. Two psychiatrists on the team reviewed the existing electronic clinical data. They retrospectively extracted information related to childhood risk factors and three adverse clinical and service outcomes (Table 1) to complete the data collecting proforma. Risk factors were chosen for their clinical relevance, their potential association with an increased risk of adverse mental health outcomes and availability to collect by clinicians as part of a service evaluation. Modifiable and non-modifiable risk factors were considered. Non-modifiable variables encompassed the level of intellectual disability, ethnicity and co-occurring diagnoses of autism and ADHD. Modifiable risk factors included challenging behaviour, psychiatric hospital admissions, mental illness, Child and Adolescent Mental Health Service (CAMHS) involvement during childhood (below 18), being a looked after child and the place of residence by the age of 16 years. Diagnoses of intellectual disability, autism, ADHD and mental illness were often made by clinicians from clinical history rather than using validated tools. Neurodevelopmental disorder diagnoses were based on DSM-5.^1^ Diagnoses of mental illnesses were made using ICD-10.^27^ Challenging behaviour was included if it was described as challenging behaviour or in similar terms in clinical records.

**Table 1:** Data collected

Adverse mental health outcome: (between age 18 and 24)	Childhood risk factors (before age of 18)
Use of psychotropic medication in the absence of severe mental illness^a^ in adulthood Cost of care packages in adulthood In-patient psychiatric admission during adulthood	Level of intellectual disability Ethnicity Diagnosis of autism Diagnosis of ADHD Challenging behaviour before the age of 18 years Psychiatric hospital admission before the age of 18 years Presence of mental illness before the age of 18 years Involvement of Child and Adolescence Mental Health Services (CAMHSs) Looked after child status before the age of 16 years Place of residence (care setting or family home) at the age of 16 years^b^

ADHD, attention-deficit hyperactivity disorder.

a. Severe mental illness – bipolar affective disorder (F31 ICD-10) and/or any psychotic disorder (F20-29 ICD-10)

b. Place of residence at the age of 16 years was used to indicate where the person lived the longest between the ages of 0 and 16 years.

### Ethics

This study constituted a service evaluation of the clinical care of the service. The National Health Service (NHS) Health Research Authority tool (http://www.hra-decisiontools.org.uk/research/index.html) confirmed that no ethical approval was required for this project. All information gathering complied with the UK Data Protection Act (2018) requirements. To ensure confidentiality, data were anonymised before analysis and handled on a service computer by authorised individuals to access the data.

### Statistical analysis

Logistic and linear regression models were employed to examine factors associated with psychotropic medication use in the absence of severe mental illness during childhood. Odds ratios with 95% confidence intervals (95% CI) are reported. Model 1 incorporated non-modifiable risk factors (level of intellectual disability, ethnicity and presence of other co-occurring neurodevelopmental disorders) as covariates. In Model 2, childhood modifiable variables (challenging behaviour, CAMHS involvement, mental illness during childhood and living setting at age 16 years) were included to examine their impact on psychotropic medication prescription while adjusting for non-modifiable risk factors.

A similar approach was adopted for modelling high-cost care packages in adulthood using multiple linear regression. The outcome variable was log-transformed to account for violation of normality and was then back-transformed for reporting purposes. All analyses were conducted using R software (version 4.3.1 for MacOS, R Core Team, Vienna, Austria; https://www.R-project.org/).^28^ The reference groups for all analyses were mild intellectual disability for the level of intellectual disability, White for ethnicity and the absence of specific conditions for other categories. We followed the approach suggested by Rothman^29^ and did not adjust for multiple comparisons.

## Results

Some 137 adults with intellectual disability aged 18–24 years were found in the whole caseload (Table 2). Among them, 27 (20.1%) had mild intellectual disability while nearly 80% had moderate to profound intellectual disability. The largest ethnic group identified was Black (45.3%) followed by White individuals (27.7%). Around 63% of the adults with intellectual disability also had a diagnosis of autism, 19% had a diagnosis of ADHD and 17% had both ADHD and autism. Some 53.3% were described as presenting with challenging behaviour. Out of those presenting with challenging behaviour, 27% had a diagnosis of ADHD and 80% had autism. Nearly half of the adults with intellectual disability had utilised CAMHSs during their childhood. Some 23 (16.9%) of the adults with intellectual disability received a diagnosis of mental illness before the age of 18 years. The majority (93.4%) lived with their families during childhood, with only a small minority in care settings. In addition, 8.8% of the adults with intellectual disability were classified as a ‘looked after child’.

**Table 2:** Cohort characteristics

Variables		*N* (%)
Total number of people with intellectual disability aged 18–24 years		137
Level of intellectual disability (missing data on level of intellectual disability – 3)	Mild	27 (20.1%)
	Moderate	67 (50.0%)
	Severe	31 (23.1%)
	Profound	9 (6.7%)
Ethnicity	White	38 (27.7%)
	Asian	9 (6.6%)
	Black	62 (45.3%)
	Mixed	7 (5.1%)
	Other unknown	21 (15.3%)
Autism	Yes	87 (63.5%)
	No	50 (36.5%)
ADHD	Yes	26 (19%)
	No	111 (81%)
Both ADHD and autism	Yes	23 (17%)
	No	114 (83%)
Challenging behaviour before the age of 18 years (missing data – 2)	Yes	72 (53.3%)
	No	63 (46.7%)
Involvement of Children and Adolescent Mental Health Services before the age of 18 years	Yes	66 (48.2%)
	No	71 (51.8%)
Diagnosis of any mental illness before the age of 18 years (missing data – 1)	Yes	23 (16.9%)
	No	113 (83.1%)
Living setting at the age of 16 years (missing data – 1)	Family	127 (93.4%)
	Care	9 (6.6%)
Categorised as a ‘looked after child’ before the age of 16 years	Yes	12 (8.8%)
	No	125 (91.2%)

ADHD, attention-deficit hyperactivity disorder.

In terms of adverse outcomes (Table 3), 28 adults with intellectual disability (20.6%) were taking psychotropic medications without a diagnosis of severe mental illness. The cost of care packages per annum varied widely, ranging from £1052 to £288 312, with a mean cost of £34 947. There were only four patients needing psychiatric hospital admission between the ages of 18 and 24 years.

**Table 3:** Adverse mental health outcomes

Use of psychotropic medications without a severe mental illness (missing data – 1)	Yes	28 (20.6%)
	No	108 (79.4%)
Cost of care package	Mean – £34 947.28	
	Range – £1052.44–288 312.40	
Psychiatric hospital admission between the ages of 18 and 24	Yes	4 (2.9%)
	No	133 (97.1%)

Table 4 presents the odds ratios, 95% CIs and *P*-values for predicting psychotropic medication prescribing without a severe mental illness in adulthood based on childhood risk factors. Model 1 indicated that the severity of intellectual disability and ethnicity did not predict psychotropic medication use. Both autism and ADHD independently predicted the risk of using such psychotropic medications. However, in Model 2, the inclusion of challenging behaviour, CAMHS involvement and mental illness resulted in ADHD and autism no longer being significantly associated with psychotropic medication prescribing. Challenging behaviour during childhood was the best predictor for psychotropic medication prescribing in adulthood (odds ratio 11.86, 95% CI 1.85–235.95, *P* = 0.028).

**Table 4:** Association between risk factors and use of psychotropic medications without a severe mental illness

	Model 1			Model 2		
	Odds ratio	95% CI	*P*-value	Odds ratio	95% CI	*P*-value
Severity of intellectual disability						
Mild intellectual disability	Ref.			Ref.		
Moderate intellectual disability	1.96	0.54–8.56	0.33	2.26	0.52–11.58	0.29
Severe intellectual disability	2.24	0.53–10.77	0.29	2.19	0.40–13.31	0.37
Profound intellectual disability	1.03	0.03–14.58	0.99	2.27	0.06–55.90	0.62
Ethnicity						
White	Ref.			Ref.		
Asian	4.03	0.62–29.36	0.15	3.02	0.40–25.53	0.29
Black	0.91	0.29–3.00	0.88	1.49	0.38–6.38	0.57
Mixed	0.90	0.10–6.50	0.92	1.94	0.12–33.78	0.63
Other & unknown	0.61	0.11–2.84	0.54	0.87	0.13–5.14	0.88
Presence of autism or ADHD						
No autism	Ref.			Ref.		
Autism	4.88	1.42–23.41	0.02	2.96	0.58–19.98	0.22
No ADHD	Ref.			Ref.		
ADHD	4.74	1.71–13.68	0.003	2.92	0.87–10.30	0.086
Modifiable factors						
No challenging behaviour				Ref.		
Challenging behaviour				11.86	1.85–235.95	0.028
No CAMHS involvement				Ref.		
CAMHS involvement				5.25	1.15–37.82	0.051
No mental illness during childhood				Ref.		
Mental illness during childhood				2.98	0.82–11.64	0.10

ADHD, attention-deficit hyperactivity disorder; CAMHS, Child and Adolescent Mental Health Service.

Table 5 displays the exp(β), 95% CIs and *P*-values for childhood risk factors predicting care package costs during adulthood. This analysis showed that autism, ADHD, increasing severity of intellectual disability and specific ethnicities initially predicted high-cost packages. However, when adjusting for challenging behaviour, CAMHS involvement, mental illness during childhood and living in a care setting in Model 2, ADHD and autism diagnoses were not significantly associated with higher cost packages. Nevertheless, intellectual disability severity and Black and mixed ethnicity status were associated with higher cost packages, while adjusting for the modifiable covariates. Furthermore, challenging behaviour emerged as an independent predictor, significantly associated with high-cost care packages compared to those who did not have challenging behaviour (exp(β) = 1.79, 95% CI 1.19–2.70, *P* = 0.006). Living in a care setting at the age of 16 years also showed an association with higher cost packages (exp(β) = −4.93, 95% CI 1.83–13.28, *P =* 0.002). Childhood mental illness and CAMHS involvement were not statistically significantly associated with high-cost care packages compared to no childhood mental illness or CAMHS involvement.

**Table 5:** Association between childhood factors and care package costs during adulthood

	Model 1			Model 2		
	exp(β)	95% CI	*P*-value	exp(β)	95% CI	*P*-value
Severity of intellectual disability						
Mild intellectual disability	Ref.			Ref.		
Moderate intellectual disability	1.97	1.23–3.17	0.005	2.08	1.34–3.22	0.001
Severe intellectual disability	2.91	1.70–4.99	0.0001	2.92	1.79–4.78	0.00003
Profound intellectual disability	3.24	1.45–7.27	0.005	4.44	2.10–9.36	0.0001
Ethnicity						
White	Ref.	Ref.				
Asian	1.03	0.49–2.18	0.93	1.26	0.63–2.51	0.51
Black	1.56	1.01–2.40	0.045	2.01	1.33–3.02	0.001
Mixed	2.56	1.05–6.26	0.039	4.29	1.89–9.78	0.0006
Other/unknown	1.34	0.77–2.33	0.30	1.80	1.08–3.00	0.03
Presence of autism or ADHD						
No autism	Ref.			Ref.		
Autism	1.57	1.07–2.32	0.02	1.24	0.84–1.83	0.28
No ADHD	Ref.					
ADHD	1.61	1.02–2.54	0.039	1.26	0.82–1.93	0.29
Modifiable factors						
No challenging behaviour				Ref.		
Challenging behaviour				1.79	1.19–2.70	0.006
No CAMHS involvement				Ref.		
CAMHS involvement				0.89	0.58–1.36	0.59
No mental illness during childhood				Ref.		
Mental illness during childhood				1.63	0.99–2.68	0.056
Living at a home setting at age 16				Ref.		
Living in a care setting at age 16				4.93	1.83–13.28	0.002
Not a looked after child				Ref.		
Looked after child				0.73	0.31–1.71	0.47

ADHD, attention-deficit hyperactivity disorder; CAMHS, Child and Adolescent Mental Health Service.

## Discussion

Even though studies have highlighted various childhood factors affecting mental health outcomes in people with intellectual disability, there is a lack of evidence on factors associated with mental health-related adverse outcomes, such as hospital admissions, psychotropic medication prescribing in the absence of severe mental illness and higher cost care packages. The present study focused on these three important clinical outcomes that are directly relevant to patients, carers, clinicians, commissioners and the wider public because of their implications for the person with intellectual disability and the care system. Out of the three outcomes, psychiatric in-patient admissions were infrequent in the group we considered and, because of this, were not included in the models.

This study demonstrated that the presence of autism and ADHD (when challenging behaviour was not adjusted for in the models) were associated with higher cost packages and the use of psychotropic medications without a severe mental illness during adulthood. To compare, studies within a non-intellectual disability context consistently show that ADHD is associated with significant functional impairments,^30,31^ persisting even after a 33-year follow-up.^32^ However, the evidence on functional impairment and long-term outcomes of ADHD in people with intellectual disability remains underexplored. Challenging behaviour has been suggested as the main functional impairment among people with ADHD and intellectual disability, with nearly 70% of adults with ADHD and intellectual disability presenting with behavioural challenges.^33^ Therefore, it can be hypothesised that challenging behaviour among people with ADHD and intellectual disability might explain the prescribing of psychotropic medications without a severe mental illness and higher cost care packages, as found in this study. Another possible explanation could be the high comorbidity of other mental illnesses, such as anxiety or mood disorders (not classed as severe mental illnesses in this study), being treated with psychotropic medications in individuals with intellectual disability co-occurring with ADHD.

Similarly, the association between autism and adverse outcomes found in this study fits in with previous studies showing a significant impact of autism on both physical and mental health in individuals with autism and intellectual disability across all ages, along with other functional impairments.^34^ Most of these impairments are observed to persist into adulthood.^35^ Furthermore, the presence of autism in people with intellectual disability is associated with challenging behaviour.^36^ The core symptoms of autism, such as repetitive and restricted behaviours and interests in children with intellectual disability, have been shown to predict challenging behaviours.^37^ Therefore, it can be hypothesised that one possible pathway linking autism to adverse outcomes in this study is through the presence of challenging behaviour. The high rate of adverse physical and mental health outcomes found in other studies may explain the significant cost of care associated with autism among people with intellectual disability.

However, these significant associations with autism and/or ADHD disappeared when challenging behaviour during childhood was accounted for in the models. It is likely that the association between ADHD and autism with these outcomes is not direct but may be mediated by challenging behaviours. To test this hypothesis, future studies require three-point longitudinal data. If confirmed, this would underscore the significance of challenging behaviour during childhood as a pivotal variable contributing to long-term adverse outcomes for people with intellectual disability. This connection may have led to increased support needs in adulthood, necessitating higher cost care packages. Furthermore, the increased reliance on psychotropic medications to manage challenging behaviour, as highlighted in previous studies,^38^ may explain the high rate of psychotropic medication use in adulthood for those with childhood challenging behaviour.

Increasing severity of intellectual disability was predictive of higher cost packages but did not significantly affect the use of psychotropic medications in the absence of severe mental illness. The increased severity of intellectual disability continued to remain a significant factor associated with higher cost packages in adulthood even after accounting for challenging behaviour. Therefore, the correlation between increasing severity and high-cost care packages in adulthood may not be mediated through challenging behaviour. Our findings align with studies that have shown that the severity of intellectual disability is not significantly associated with increased challenging behaviour,^37^ but this contrasts with studies showing the opposite: that increasing severity of intellectual disability predicts challenging behaviour.^39^ The higher cost associated with more severe intellectual disability found in this study might simply be attributed to increasing support needs of people with more severe intellectual disability because of low levels of adaptive behaviour skills and limited communication skills.

The role of ethnicity in terms of adverse outcomes was also explored in this study. Studies in non-intellectual disability populations have shown that Black and minority ethnic people are more likely to receive a diagnosis of mental illness compared to White people.^40^ Our findings showed that, compared to White people, people with intellectual disability from Black and mixed ethnic backgrounds were more likely to need higher cost packages. However, ethnicity did not play a significant role in psychotropic medication prescribing, even after accounting for challenging behaviour, CAMHS involvement and mental illness during childhood. Studies have shown that people with intellectual disability from Black minority ethnic backgrounds experience worse health outcomes compared to people with intellectual disability from White ethnic backgrounds, owing to indirect causes such as reduced healthcare utilisation.^41^ Deprivation may be an additional factor contributing to adverse mental health outcomes among people with intellectual disability from ethnic minority groups.^36^ The predominance of Black ethnicity in the study sample might have influenced these findings. However, these results raise the question of whether targeted interventions are needed for children with intellectual disability from ethnic minority backgrounds.

Overall, the key finding highlights the significant association between the presence of challenging behaviour during childhood and both higher cost care and increased psychotropic prescribing in the absence of severe mental illness. It highlights the nature and severity of challenging behaviour, serving as a stark reminder of the important need for various strategies to support and reduce behavioural challenges in children with intellectual disability from an early age.

### Implications

This study established the importance of exploring clinically important outcomes in addition to caseness and mental health symptomatology, which have often been investigated in existing studies. Such outcomes are important to individuals with intellectual disability and their carers in addition to service providers and commissioners. Factors significantly associated with adverse outcomes in adulthood that are identified in this study need further analysis to determine causation. Therefore, direct claims cannot be made about their importance, but this study highlights their associations with adverse outcomes in young adulthood. The findings of this study may help to highlight the importance of challenging behaviour, which is a presentation rather than a diagnosis itself in long-term outcomes, as well as underlying neurodevelopmental disorders such as ADHD, which may drive challenging behaviour. Understanding the importance of associated risk factors may help to actively look for co-occurring neurodevelopmental disorders and develop appropriate screening and diagnostic tools to identify those with neurodevelopmental disorders when people with intellectual disability access mental health services and overcome diagnostic invisibility among children with intellectual disability. The findings underscore the importance of early intervention for prevention or reduction of challenging behaviours in children with intellectual disability and a likely particular focus on children with intellectual disability and additional neurodevelopmental disorders, such as autism and ADHD.

### Limitations

The study's retrospective design suggests that childhood risk factors relied on accurate documentation by clinicians. ADHD is often underdiagnosed among people with intellectual disability, and therefore the number of children with ADHD in this sample may be an underestimation. Only a limited number of risk factors were explored in this study. Numerous risk factors have been shown to contribute to adverse health outcomes in the general population, such as trauma and adverse childhood experiences, but these were not included here because of challenges with retrospectively collecting data from childhood records. Generalisability is limited because of sampling from a single intellectual disability service but participants were ethnically diverse, with most children growing up in relatively deprived households. Finally, wide confidence intervals for challenging behaviour as a predictor of psychotropic medication prescribing suggest measurement instability, which may be because of the small sample size or measurement error.

This study highlighted several significant predictors of adverse outcomes. These risk factors need to be considered by services supporting individuals with intellectual disability. Developing early detection tools for individuals at ultra-high risk for later adverse mental health outcomes in people with intellectual disability is important to reduce the gap in mental health disparities. It is likely to enhance clinical decision-making to expedite preventative interventions during childhood to ensure improved positive future opportunities and increased quality of life.

## Data Availability

The data are not publicly available because of information within the data that could compromise the privacy of participants in this study.
