# Childhood risk factors and clinical and service outcomes in adulthood in people with intellectual disabilities – CORRIGENDUM

**DOI:** 10.1192/bjo.2025.15

**Published:** 2025-02-03

**Authors:** B. Perera, S. Mufti, C. Norris-Grey, A. Baksh, V. Totsika, A. Hassiotis, P. Hurks, T. van Amelsvoort

This article was originally published with co-author Caitlin Norris-Grey’s name incorrectly given as ‘C. Norris’.

This has now been updated and this corrigendum published.
